# Physiologic Impact of Circulating RBC Microparticles upon Blood-Vascular Interactions

**DOI:** 10.3389/fphys.2017.01120

**Published:** 2018-01-12

**Authors:** Ahmed S. Said, Stephen C. Rogers, Allan Doctor

**Affiliations:** ^1^Department of Pediatrics, Washington University in St. Louis, St. Louis, MO, United States; ^2^Biochemistry and Molecular Biophysics, Washington University in St. Louis, St. Louis, MO, United States

**Keywords:** erythrocytes, nitric oxide, endothelium, vasoregulation, blood flow, red blood cells, microparticle

## Abstract

Here, we review current data elucidating the role of red blood cell derived microparticles (RMPs) in normal vascular physiology and disease progression. Microparticles (MPs) are submicron-size, membrane-encapsulated vesicles derived from various parent cell types. MPs are produced in response to numerous stimuli that promote a sequence of cytoskeletal and membrane phospholipid changes and resulting MP genesis. MPs were originally considered as potential biomarkers for multiple disease processes and more recently are recognized to have pleiotropic biological effects, most notably in: promotion of coagulation, production and handling of reactive oxygen species, immune modulation, angiogenesis, and in initiating apoptosis. RMPs, specifically, form normally during RBC maturation in response to injury during circulation, and are copiously produced during processing and storage for transfusion. Notably, several factors during RBC storage are known to trigger RMP production, including: increased intracellular calcium, increased potassium leakage, and energy failure with ATP depletion. Of note, RMP composition differs markedly from that of intact RBCs and the nature/composition of RMP components are affected by the specific circumstances of RMP genesis. Described RMP bioactivities include: promotion of coagulation, immune modulation, and promotion of endothelial adhesion as well as influence upon vasoregulation via influence upon nitric oxide (NO) bioavailability. Of particular relevance, RMPs scavenge NO more avidly than do intact RBCs; this physiology has been proposed to contribute to the impaired oxygen delivery homeostasis that may be observed following transfusion. In summary, RMPs are submicron particles released from RBCs, with demonstrated vasoactive properties that appear to disturb oxygen delivery homeostasis. The clinical impact of RMPs in normal and patho-physiology and in transfusion recipients is an area of continued investigation.

## Microparticles (MP) overview

**Figure 1:**
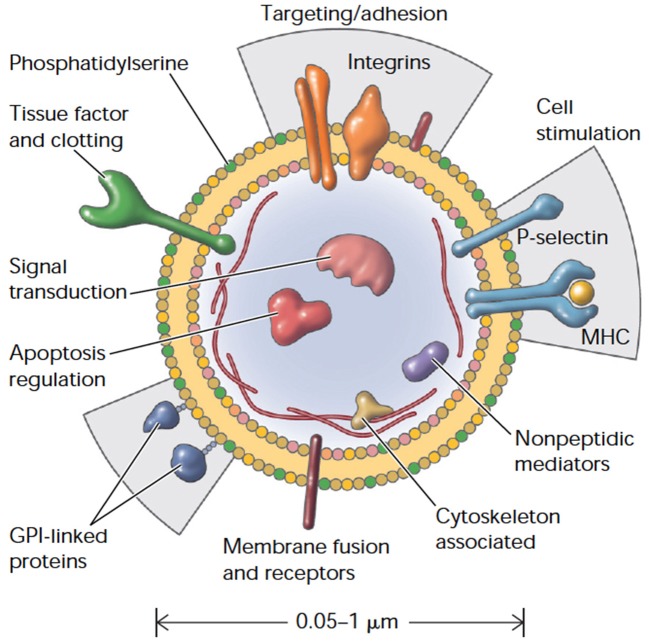
Cellular microparticles: a mobile storage pool of bioactive effectors. Membrane microparticles are shed from the plasma membrane of stimulated cells, harboring cytoplasmic proteins as well as bioactive lipids implicated in a variety of fundamental processes. MHC, Major histocompatibility complex; GPI, glycosylphosphatidylinositol. Adapted with permission from Hugel et al. (2005).

**Figure 2:**
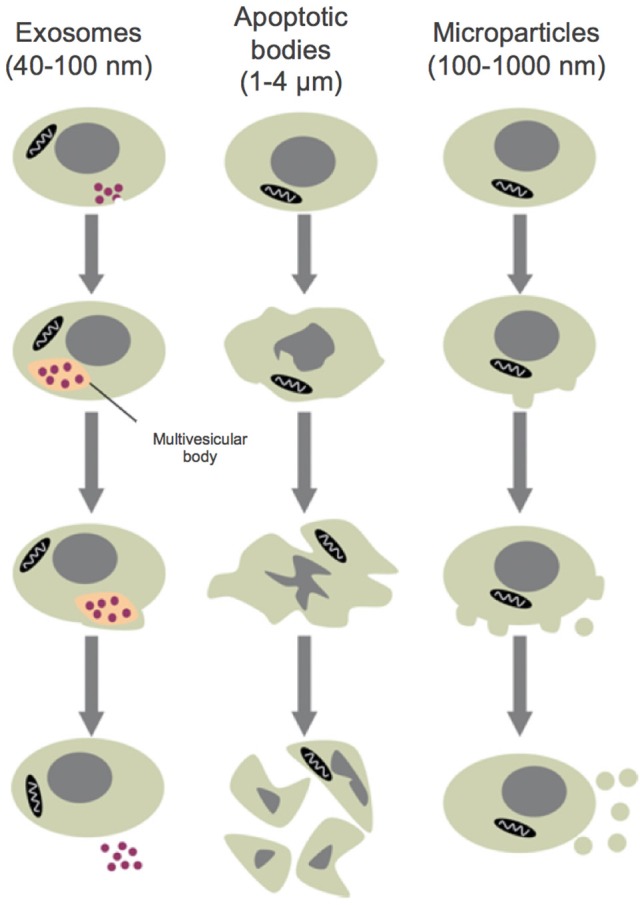
Size classes of extracellular vesicles. Exosomes are formed through inward membrane budding, leading to formation of 40–100 nm intracellular vesicles which accumulate within multivesicular bodies that are subsequently released to the extracellular milieu. Apoptotic bodies may contain DNA and/or organelles and are formed during the late stages of apoptosis, after cell shrinkage. Microparticles are formed from the outward blebbing of membrane and released into the extracellular space. Adapted with permission from Burger D. et al. (2013).

Genesis of small membrane-encapsulated vesicles (termed microparticles, MPs) from activated and/or apoptotic cells was first reported ~40 years ago (Boulanger and Dignat-George, 2011). Formally defined, MPs are cell-derived vesicles that are 0.1–1.0 μm in size and are categorized by membrane proteins and cytosolic material that is specific to various parent cell populations (Morel et al., 2011; Figure 1). MPs are distinguished from exosomes and apoptotic bodies by size, composition and mechanism of formation (Burger D. et al., 2013; Figure 2). Exosomes are generally smaller (40–100 nm) and form by a multistep process that involves intracellular generation and subsequent vesicle extrusion; apoptotic bodies are much larger (1–5 μm) and arise via shedding during apoptosis (Elmore, 2007).

### Cell-cell communication via MPs:

MPs serve as vehicles for inter-cellular exchange of biological material and information, for which two principle mechanisms have been proposed: (1) MPs act as circulating modules for complex signaling, activating receptors on target cells by presenting organized clusters of membrane-associated bioactive molecules (Del Conde et al., 2005), and/or (2) direct transfer of MP contents, including proteins, bioactive lipids or RNA to recipient cells—thereby, promoting phenotypic modification and reprogramming of cell functions (Whale et al., 2006). As such, MP based cell-cell communication enables a unique form of remote signaling from MP-parent to target cells, by presenting a complex array of ligands for membrane receptors, paired with a concentrated payload of bioactive molecules and substrate for intracellular delivery (Mause and Weber, 2010).

### MPs as disease biomarkers

MP formation is enhanced by stress and injury and consequently, MPs have been considered as potential biomarkers for disease onset and progression. As such, it is important to recognize that moment-specific abundance of circulating MPs is determined by the balance between MP formation and clearance (e.g., MP abundance and flux do not necessarily correlate and, high-flux low-abundance states may have physiologic significance that is belied by measured MP level, alone.). MP levels, in particular: for platelet-, leukocyte-, and endothelium-related MPs, are known to increase in the setting of vascular injury, pro-thrombotic and pro-inflammatory states that complicate a broad array of health conditions, such as diabetes (Feng et al., 2010), pulmonary hypertension (Forest et al., 2010), chronic kidney disease (Faure et al., 2006), preeclampsia (González-Quintero et al., 2003), atherosclerosis (Bernal-Mizrachi et al., 2003), and heart failure (Amabile et al., 2012) amongst others.

### MP formation

**Figure 3:**
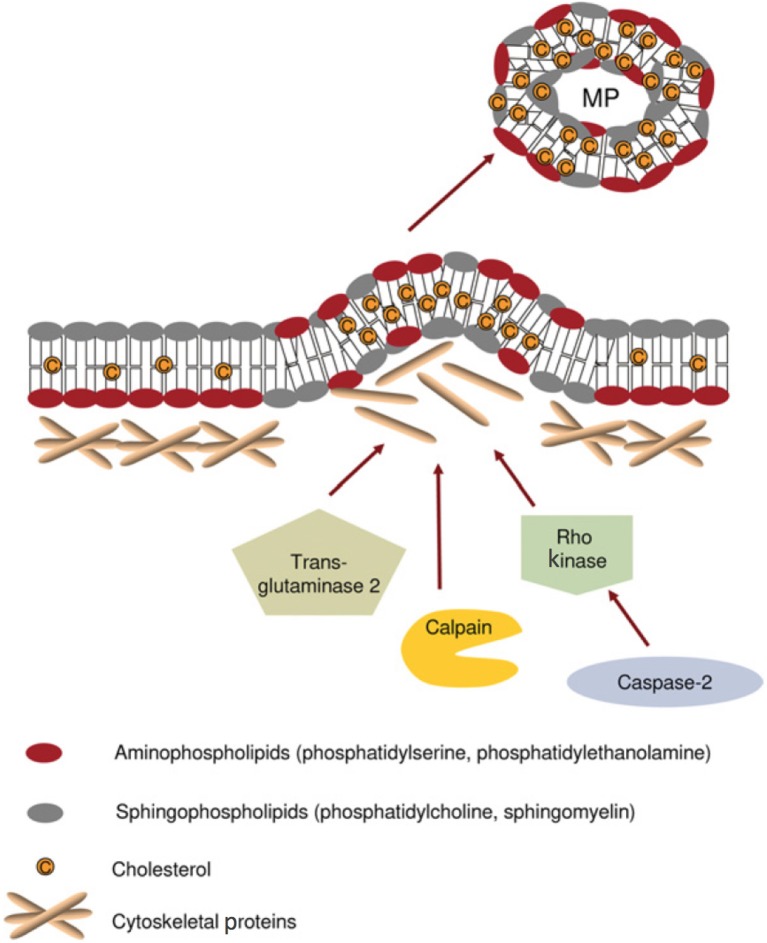
Mechanisms proposed for cytoskeleton remodeling leading to microparticle (MP) formation. Under normal conditions, aminophospholipids (phosphatidylserine and phosphatidylethanolamine) are found exclusively on the inner leaflet of the plasma membrane. During MP formation, membrane asymmetry is lost as aminophospholipids redistribute to the outer leaflet of the plasma membrane. Cytoskeletal re-organization results in the outward blebbing of the plasma membrane and may be dependent upon actin polymerisation, caspase 2/Rho kinase, calpain, and/or transglutaminase 2. Such processes may vary between different cell types. MP formation appears to occur selectively in lipid-rich microdomains (lipid rafts/caveolae) within the plasma membrane. Adapted with permission from Burger D. et al. (2013).

MPs arise from diverse cell types, including vascular elements (endothelial and vascular smooth muscle cells) (Rautou et al., 2011), blood components [erythrocytes (Tissot et al., 2010), platelets and leukocytes], cardiomyocytes (Antoniak et al., 2009) and podocytes (Burger D. et al., 2013), as well as various cancers (Zahra et al., 2011) and progenitor cell populations (Chen et al., 2010). MPs form via outward blebbing and shedding of the plasma membrane (Dignat-George and Boulanger, 2011). This poorly understood process appears to involve two main steps: (1) an initial cytoskeletal re-organization (Cauwenberghs et al., 2006), involving actin filament rearrangement that appears initiated by activation of calpain (Nolan et al., 2008), rho kinase (Sapet et al., 2006), and transglutaminase (van den Akker et al., 2012; Figure 3) and (2) externalization of phosphatidylserine (PS), a negatively charged aminophospholipid found almost exclusively on the plasma membrane inner leaflet, in healthy cells (Bevers et al., 1999). In red blood cells, PS “sidedness” is controlled by an ATP/calcium dependent system involving three distinct enzymes: flippase, floppase, and scramblase (Kostova et al., 2015). Of note, defective PS externalization underlies Scott syndrome, a bleeding disorder associated with diminished platelet MP shedding (Leroyer et al., 2009).

**Figure 4:**
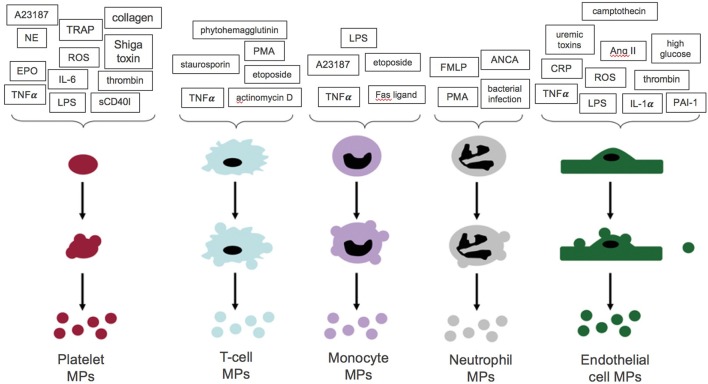
Stimuli for microparticle (MP) formation from platelets, endothelial cells, and leukocytes. A summary of the stimuli which promote MP formation from platelets, endothelial cells and leukocytes. NE, Norepinephrine; EPO, erythropoietin; sCD40I, soluble CD40 ligand. With permission from Burger D. et al. (2013).

Studies of cultured cells have identified several stimuli for MP formation, including: various hormones, fatty acids, reactive oxygen species (e.g., hydrogen peroxide) (Aoki et al., 2007) as well as increased intracellular calcium (Fox et al., 1991). Activation of several surface receptors has also been shown to drive MP production, such as by tumor necrosis factor (TNF)-α (Eyre et al., 2011) on monocytes, leukocytes, and neutrophils, as well as by pro-inflammatory [lipopolysaccharide (Ståhl et al., 2011), shiga toxin (Ståhl et al., 2011), and cytokines (Nomura et al., 2000)] and pro-coagulant ligands [thrombin (Terrisse et al., 2010), collagen (Takano et al., 2004), and norepinephrine (Tschuor et al., 2008)] on platelets and Toll-like receptor 4 on dendritic cells (Théry et al., 2009; Figure 4).

### MP clearance

MP elimination via the mononuclear phagocyte system (MPS) appears to regulate circulating MP availability for target-cell fusion; however, less is known about this process than about MP formation (Burger D. et al., 2013). Macrophages ingest co-cultured MPs and externalized PS is thought to activate scavenger receptors, promoting MP endocytosis (Terrisse et al., 2010). MP surface IgM has also been shown to promote MP clearance by macrophages (Litvack et al., 2011).

### MP-mediated effects upon vascular physiology

Although MP shedding may be enhanced by stress, exocytosis is a constitutive process for the majority of blood and vascular cell types (Angelillo-Scherrer, 2012). Depending on the stimulus, however, protein content (of both “cytoplasm” and membrane) for MPs derived from the same cell lineage can vary (Jimenez et al., 2003). Moreover, the enzymes that govern MP shedding can be selective, depending on the activating agonist and/or parent cell microenvironment (Peterson et al., 2008; Bernimoulin et al., 2009). Such tight regulation of MP production suggests MPs may facilitate important cell-cell communication in a context-specific fashion. Of note, MPs are internalized by a variety of cells (macrophages and endothelial cells, amongst others) in a dose-dependent manner, enabling “MP cargo” transfer between cells in a fashion that influences target cell function and phenotype (Diehl et al., 2012).

The most well-characterized components of vascular physiology that are influenced by MPs include the following:

#### Coagulation

This is perhaps the most clearly established example of MP-based influence. Platelet derived MPs have effects similar to activated platelets in initiation of thrombin generation and clot propagation, despite having at least two-orders-of-magnitude difference in surface area (Sinauridze et al., 2007). Moreover, externalized phospholipids (mainly PS) create a negatively charged surface that anchors cationic domains of proteins involved in assembly of the multi-component (tenase) complex that leads to the thrombin burst (Owens and Mackman, 2011).

#### Oxidative stress

MPs of differing derivations, produced under various stimuli, have been shown to affect the enzymatic systems controlling reactive oxygen species generation. Both endothelial and monocyte derived MPs are known to increase superoxide (Mastronardi et al., 2011) and hydrogen peroxide production (Burger et al., 2012) as well as to uncouple nitric oxide synthase (NOS) (Essayagh et al., 2007). However, activated T-cell related MPs have been shown to dampen reactive oxygen species production and to increase nitric oxide (NO) production (Agouni et al., 2007).

#### Inflammation

Pro-inflammatory signaling generally provokes MP shedding and, in an amplifying signaling loop, MPs may directly contribute to the inflammatory response (e.g., PMN derived MPs promote endothelial IL-6 and monocyte chemotactic protein release) (Mesri and Altieri, 1999). MPs are also thought to promote inter-cellular inflammatory cell interaction and adhesion; specifically, endothelial-derived MPs increase adhesion molecule expression and facilitate monocyte-endothelial cell interactions (Burger D. et al., 2011), in addition to binding to monocytes and promoting transendothelial migration (Jy et al., 2004).

#### Angiogenesis

Platelet-derived MPs have been implicated in regulation of angiogenesis. In rats following myocardial ischemia, platelet MPs increase post-ischemic capillary density, and proliferation (Brill et al., 2005) and are reported to promote tube formation by human umbilical vein endothelial cells (Kim et al., 2004). This is not surprising, as platelets are known to contain at least 20 angiogenesis regulating factors. Moreover, certain stimulated T-cell related MPs have been shown to inhibit angiogenesis both *in vivo* and *in vitro* (Yang et al., 2012).

#### Apoptosis

Endothelial- and monocyte-derived MPs are described to promote cellular senescence and apoptosis in circulating angiogenic and endothelial progenitor cells, respectively (Huang et al., 2010; Distler et al., 2011). This process appears linked to phagocytosis of MPs that contain high amounts of membrane arachidonic acid, leading to caspase activation and initiation of apoptosis (Huber et al., 2007).

## Red blood cell (RBC) derived microparticles (RMPs)

RBC-derived MP formation occurs routinely during normal maturation *in vivo*; *ex vivo*, this process is accelerated by processing and storage, prior to transfusion (Greenwalt, 2006). RMPs are generally smaller than MPs of other origin, are more homogenous in size (~0.15 μm in diameter), and are often accompanied by smaller vesicles, termed nanovesicles (Allan et al., 1980). During their 120-day lifespan, RBCs lose ~20% of their volume through vesicle emission, increasing intra-erythrocytic Hb concentration by ~14%; metrics for RMP production, circulating number and volume are presented in Table 1 (Willekens et al., 2008). It was originally thought that vesiculation served to rid RBCs (which lack lysosomes) of damaged or harmful components that might otherwise accumulate over time, such as denatured Hb, C5b-9 complement attack complexes, or Band 3 neoantigens (Bosman et al., 2008b). It has also been suggested that RMP shedding promotes recognition and clearance of senescent and/or damaged RBCs by removing integral self-marker membrane proteins (e.g., CD47) (Stewart et al., 2005).

**Table 1:** Estimated total circulating number, volume and rate of production of RMPs, intact RBCs and their respective ratios in a healthy adult male (Willekens et al., 2008).

	**Total circulating number**		**Volume per RMP/RBC**		**Rate of production**	
	**RMP**	**RBC**	**RMP**	**RBC**	**RMP**	**RBC**
Absolute number	8.5 × 10^8^	2.5 × 10^13^	0.065 μm^3^	88 μm^3^	5.8 × 10^8^/s	1.4 × 10^6^/s
RMP: RBC ratio	3.4 × 10^−5^:1		7.38 × 10^−4^:1		8,120:1	

### RMP production

RBCs spontaneously shed PS-positive MPs (Burger P. et al., 2013) and each individual RBC is estimated to generate ~230 vesicles during it's lifespan (Bosch et al., 1994). As for other cell types, membrane phospholipid rearrangement is an integral step in RMP formation. The normal asymmetric distribution of the lipid bilayer is controlled by 3 different elements; flippase (ATP-dependent enzyme that promotes inward orientation of negatively charged lipids), floppase (responsible for maintaining outward orientation of phosphatidylcholine) and scramblase (facilitating bidirectional movement of all phospholipids) (Daleke, 2003). Consequent to RBC injury, metabolic stress, and/or senescence (and storage), ATP depletion and potassium leakage diminish flippase activity, while elevated intracellular calcium increases scramblase activity; consequently, normal membrane asymmetry is lost, PS is exposed on the RBC surface and vesicle shedding is promoted (Burger P. et al., 2013). There is lack of consensus with regard to the RBC sub-population most responsible for RMP production. Some suggest that senescent RBCs are responsible for the majority of RMP production *in vivo* (Willekens et al., 2003), while others have shown that during storage (or other periods of metabolic stress), younger RBC sub-populations produce the majority of RMPs (Greenwalt, 2006). Of note, during storage, RMP composition/content may vary with specific conditions and duration (Piccin et al., 2015). For example, RMPs generated *in vitro*, by stimulation with Ca ionophores, differ in size and cytoskeletal protein structure than RMPs generated during RBC storage (Allan et al., 1980; Salzer et al., 2002). Additionally, RMPs isolated during storage have less variation in size and shape than those isolated from circulation (Greenwalt, 2006). Moreover, hypotonic, alkaline storage solutions are associated with increased RMP production and with an RMPs that have diminished cholesterol, phospholipids, as well as band 3 and protein 4.1 (Greenwalt, 2006). Leukoreduction diminishes RMP production by up to 40–50%, particularly under anaerobic conditions (Jy et al., 2012). More generally, RBC storage (and other injury states) is characterized by progressive depletion of energy resources and antioxidant defenses, enabling accumulation of oxidative modification to proteins (and lipids), particularly involving the cytoskeleton and Band 3 (Kriebardis et al., 2008). Vesiculation may enable elimination of such markers as well as other dysfunctional elements that accumulate during storage, or consequent to *in vivo* injury (Delobel et al., 2012).

### Triggers for RMP production

Little is known about the specific signaling that regulates RBC vesiculation, both during RBC aging *in vivo* and during *ex vivo* storage. Further, it is also important to recognize that unique changes may occur to stored RBCs *in vivo*, following transfusion. Several RMP production triggers have been identified, mostly linked to (but not unique to) the changes RBCs undergo during storage. These include:

- *Increased cytosolic calcium (Ca*^*2+*^*)* is the most well-characterized trigger for activation of Ca^2+^ dependent proteases, leading to cytoskeletal damage and activation of Ca^2+^ dependent scramblase; both processes are result in PS exposure and MP shedding (Bevers et al., 1999).
- *ATP depletion* impairs performance of the major ATP-dependent transporter proteins (flippase and floppase) responsible for maintaining cell membrane asymmetry; loss of asymmetry promotes MP budding and shedding (Burger P. et al., 2013).
- *Increased potassium (K*^+^*) leakage* has also been linked to disturbed erythrocytic membrane transporter activity, disturbing maintenance of membrane structure and leading to MP formation (Burger P. et al., 2013).
- *Other cascades arising from energy failure* in RBCs have been shown to increase RBC vesiculation and MP formation. These include G protein-coupled receptor signaling, the phosphoinositide 3-kinase (PI3K-Akt protein kinase B) pathway, the Jak-STAT (Janus kinase-signal transducer and activator of transcription) pathway and the Raf-MEK (mitogen-activated protein kinase)-ERK (extracellular signal-regulated kinase) pathway (Kostova et al., 2015).

### RMP ~ RBC differences

Proteomic analysis demonstrates that RMP protein content is diverse, including carbonic anhydrase, peroxiredoxins, and 14-3-3 proteins (regulators of a number of processes, such as protein kinase activity and signal transduction) (Rubin et al., 2008). RMPs, however, are structurally and functionally different from intact RBCs (i.e., RMPs are not “just small RBCs”). As discussed above, the RMP shedding process involves loss of normal membrane asymmetry, with increased density of negatively charged phospholipids (e.g., PS) on the outer RMP membrane (leading to differing surface potential). In comparison to parent RBCs, RMP membranes are also enriched with specific proteins (Band 3 dimers) (Bosman et al., 2008b). Moreover, in comparison to intact RBCs, storage-related RMPs appear enriched with stomatin, relatively depleted in actin and to have more stable glycophorin A (Rubin et al., 2008). Disruption of normal cytoskeletal protein structure (de Jong et al., 1996) is also integral to RMP shedding (Rubin et al., 2012) and distinguishes daughter from parent cells. These structural characteristics, in addition to size discrepancy, result in important differential “streaming” between RBCs and RMPs, with preferential RMP circulation in proximity to endothelial cells in the “cell free” zone of the micro-circulation (Liu et al., 2013) (N.B. This feature has significant physiologic implications, vide infra.). Finally, RMPs encapsulate a significant amount of Hb (Greenwalt et al., 1991), which confers physiologic characteristics closer to cell free Hb than to intact RBCs [particularly with regard to interactions with nitric oxide (NO)].

### RMP effects upon vascular physiology

There is increasing recognition of RMP biological effects, particularly in the context of transfusion. Proposed effects include the following:

#### Promotion of coagulation

It is well-established that negatively charged surfaces activate the zymogen components of the coagulation cascade, and it appears that RMPs promote coagulation in this fashion. In the presence of low exogenous tissue factor, RMPs increase thrombin generation and remarkably, are capable of initiating and propagating thrombin generation even in the absence of tissue factor (Rubin et al., 2013). There is some evidence that this pro-coagulant activity is dependent on Factor XII (Van Der Meijden et al., 2012). Some authors have suggested that tissue factor may be present on the RMP surface, contributing to their pro-coagulant effect (Biró et al., 2003). Of note, RMP abundance is known to increase in certain hypercoagulable states associated with hypercoagulability, such as sickle cell crises (van Beers et al., 2009). Conversely, RMPs also bind protein S, a cofactor for activated protein C, which enhances degradation of Factors VIIIa and Va, and inhibits tenase and prothrombinase and thus, promotes clot lysis (Koshiar et al., 2014). As such, depending on context, balance amongst these pleotropic effects determines RMP “coagulation phenotype (Koshiar et al., 2014).”

#### Nitric oxide (NO) scavenging

Nitric oxide (NO) is a vasodilator effector component of physiologic reflexes that subserve dynamic matching between regional blood flow and tissue respiration (Doctor and Stamler, 2011). Of note, extra-erythrocytic hemoglobin (Hb) reacts with NO in a diffusion-limited oxidation reaction that quenches NO bioactivity, disrupting vasoregulation and oxygen delivery homeostasis; under normal conditions, this effect is limited by Hb compartmentalization within RBCs (Singel and Stamler, 2005). Specifically, constraints upon Hb~NO interaction are substantially influenced by RBC size and membrane architecture (Huang et al., 2001); however, reaction between de-compartmentalized, cell-free Hb, and NO lacks such constraint (Lancaster, 1994). Notably, RMP:NO interaction more closely mirrors that of cell-free Hb than that of intact RBCs, and as such—RMPs act as potent NO scavengers [RMP reaction with NO is ~1,000-fold faster than with RBC-encapsulated Hb (Donadee et al., 2011) and is only 2.5- to 3-fold slower than with cell-free Hb (Donadee et al., 2011)]. The potential impact of quenching NO bioactivity by RMPs (following transfusion) dramatically increases with the increase in RMP abundance that is observed during storage duration. Of note, the degree by which RMPs influence NO bioavailability *in vivo* is dependent on several factors, most importantly, the degree to which RMPs enter the cell-free zone in the microcirculation (e.g., stream in immediate proximity to endothelium) (Liu et al., 2013).

#### Immune modulation

Transfusion Related Immune Modulation (TRIM) is a recognized, but poorly characterized, complication of transfusion. Given the known increase in RMP generation with storage duration, a role for RMPs in TRIM pathobiology (which appears linked to RBC unit age) has been postulated (Muszynski et al., 2017). RMPs influence antigen presenting cells (APC) and boost mitogen driven T cell responses (Danesh et al., 2014); specifically, RMPs amplify APC-based induction of pro-inflammatory cytokines and chemokines from peripheral blood mononuclear cells (PBMC), promoting their survival (pro-inflammatory effect) (Danesh et al., 2014). Alternatively, RMPs may exert an immunosuppressive effect, by dampening release of various cytokines such as TNF-α, IL-8, or IL-10 (Sadallah et al., 2008). Of note, production of sickle cell derived RMP (SS RMPs) is enhanced during inflammation; moreover, when engulfed by myeloid cells, SS RMPs promote pro-inflammatory cytokine secretion and endothelial cell adhesion, suggesting that crosstalk between circulating inflammatory cells and SS RMPs contributes to sickle cell disease (SCD) pathogenesis (Awojoodu et al., 2014). Additionally, since RMPs have also been demonstrated to bear blood group antigens (Canellini et al., 2012); transfusion-related RMPs may therefore represent a significant immunogenic load (Willekens et al., 2008) and contribute to the severity of alloimmunization in chronically transfused patients (Canellini et al., 2012).

#### Promotion of endothelial adhesion

As noted above, RMPs appear to play a significant role in the pathophysiology of SCD, during which RMP production is enhanced, promoting pro-inflammatory cytokine secretion (van Beers et al., 2009). SS RMPs have also been shown to enhance adhesion of intact RBCs to endothelial cells (Awojoodu et al., 2014). Of note, up to a third of the circulating extra-erythrocytic heme in patients with SCD may be carried by RMPs (Camus et al., 2015); moreover, externalized membrane PS on SS RMPs retains heme on the external RMP surface; such heme-laden SS RMPs transfer heme directly to vascular endothelium, generating oxidative stress and endothelial apoptosis (Camus et al., 2015). This linkage of hemolysis to endothelial injury has been proposed as a trigger for SCD vaso-occlusive crises.

### RMP clearance

As for other MP populations, RMPs are likely to be cleared via the MPS, by hepatic Kupffer cells (Willekens et al., 2005). Such removal appears to occur very rapidly and is thought to be mediated by PS-binding scavenger receptors and senescent cell antigen specific autoantibodies (Willekens et al., 2005). Immunologic analysis of RMPs elaborated by senescent RBCs demonstrates Band 3 clustering; this RMP subpopulation may therefore be cleared in a fashion similar to that for intact senescent RBCs (Willekens et al., 2008).

### RMPs and RBC processing and storage for transfusion

**Figure 5:**
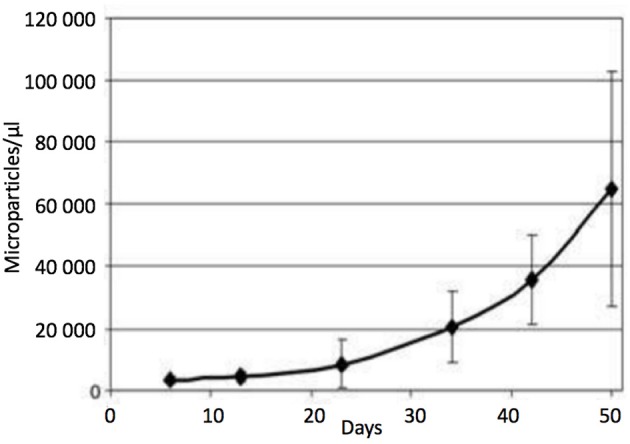
Microparticle (MP) count in red blood cell units during storage (without centrifugation). Data are expressed as the mean *SD* (*n* = 7). At day 5: 3,371,188 MPs/μL were counted, whereas at day 50: MPs had increased in abundance by ~20-fold. MPs were stained with anti-human CD47. With permission from Rubin et al. (2008).

The RBC storage lesion is comprised by profoundly altered metabolism and biomechanics; in particular, energy failure (with impaired ATP and reducing equivalent production) is characteristic and is associated with a significant acidosis, decrease in 2,3-diphosphoglycerate and failure of the membrane Na^+^/K^+^ ATPase pump with continuous potassium leakage (Bennett-Guerrero et al., 2007; D'Alessandro et al., 2015) (e.g., conditions known to promote RMP genesis; Bosman et al., 2008a; Karon et al., 2009). Morphologically, stored RBCs slowly morph from smooth biconcave discs, to spiculated echinocytes, to dense sphero-echinocytes; these changes arise from membrane loss and RMP formation, and lead to loss of cell volume regulation and diminished deformability (Greenwalt, 2006), thus negaitvely impacting post-transfusion survival and rheology (Relevy et al., 2008). In fact, RMP genesis appears directly proportional to storage duration, with a 20-fold increase after 50 days (Rubin et al., 2008; Figure 5); the doubling time for RMP concentration during storage is estimated to be 9 days (95% CI: 7.7–10.7 days) (Donadee et al., 2011). The specific reasons for such significant RMP production remain unknown; it has been suggested that storage activates a physiologic process that serves (*in vivo*) as means to prevent premature RBC clearance, by shedding membrane proteins that would otherwise signal RBC senescence (Solheim et al., 2004; Willekens et al., 2008).

## Physiologic impact of storage-generated RMPs during RBC transfusion

### Oxygen (O_2_) delivery homeostasis and vasoregulation

**Figure 6:**
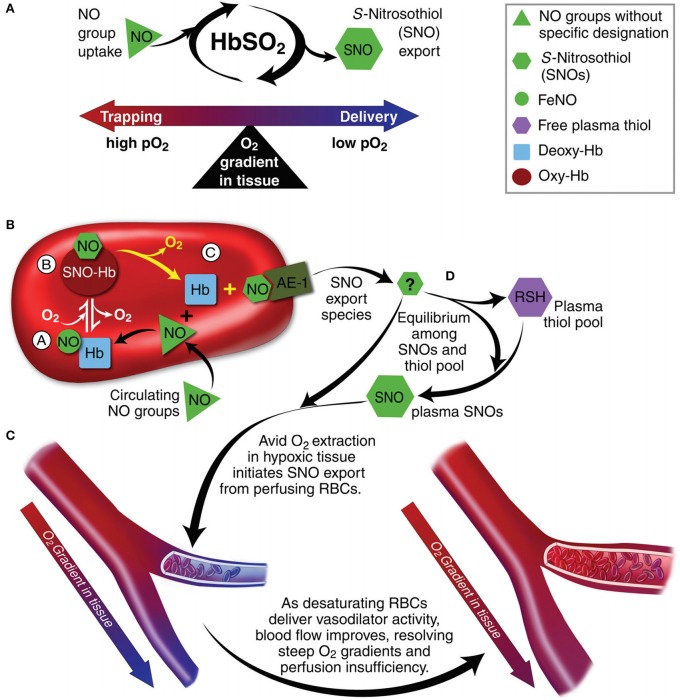
Red blood cells (RBC) transduce regional O_2_ gradients in tissue to control nitric oxide (NO) bioactivity in plasma by trapping or delivering NO groups as a function of hemoglobin (Hb) O_2_ saturation (Hb SO_2_). **(A)** Circulating NO groups are processed by Hb into the highly vasoactive (thiol-based) NO congener, S-nitrosothiol (SNO). By exporting SNOs as a function of Hb deoxygenation, RBCs precisely dispense vasodilator bioactivity in direct proportion to the lack of regional blood flow. **(B)** O_2_ delivery homeostasis requires biochemical coupling of vessel tone to environmental cues that match perfusion sufficiency to metabolic demand. Because oxygenated Hb (oxy Hb) and deoxygenated Hb (deoxy Hb) process NO differently, allosteric transitions in Hb conformation afford context-responsive (O_2_-coupled) control of NO bioavailability, thereby linking the sensor and effector arms of this system. Specifically, Hb conformation governs the equilibria among **(A)** deoxygenated Hb FeNO (NO sink), **(B)** oxygenated SNO-Hb (NO store), and **(C)** acceptor thiols including the membrane protein SNO-AE-1 (bioactive NO source). Direct SNO export from RBCs or S-transnitrosylation from RBCs to plasma thiols **(D)** yields vasoactive SNOs, which influence resistance vessel caliber and close this signaling loop. Thus, RBCs either trap **(A)** or export **(D)** NO groups to optimize blood flow. **(C)** NO processing in RBCs **(A,B)** couples vessel tone to tissue pO2; this system subserves hypoxic vasodilation in the arterial periphery and thereby calibrates blood flow to regional tissue hypoxia. Adapted with permission from Doctor and Stamler (2011).

Tissue O_2_ delivery is a function of blood O_2_ content and regional blood flow, with the latter being the principle determinant. As such, the volume and distribution of regional blood flow is actively regulated to maintain dynamic coupling between O_2_ delivery and tissue respiration (Kulandavelu et al., 2015). It is now commonly appreciated that RBCs act as both sensors and transducers, comprising a signaling loop in this physiology, by linking bioavailability of vasoactive effectors in plasma to microcirculatory O_2_ gradients (and thereby, modulating resistance vessel caliber to maintain perfusion sufficiency) (Doctor and Stamler, 2011). This key physiologic reflex is termed hypoxic vasodilation (HVD) and is primarily mediated by RBC-transported NO (Gonzalez-Alonso et al., 2001; McMahon et al., 2002; Doctor et al., 2005); as such, by serving as HVD effector elements, RBCs function as a key node in maintenance of O_2_ delivery homeostasis (Figure 6). This paracrine RBC function is governed by O_2_-linked transitions in Hb conformation which (because of differing reactions of deoxy- and oxy-Hb with NO) transduce regional pO_2_ gradients into NO bioactivity, thereby effecting vasodilation to resolve perfusion insufficiency (e.g., HVD) (Singel and Stamler, 2005). Of note, this physiology is disrupted when RBCs release Hb into plasma. Specifically, although Hb packaging in RBCs blunts NO consumption ~1,000-fold, once released, free Hb (and RMP contained Hb) readily inactivates NO, preventing facile NO-based traffic between RBCs and endothelium (Vaughn et al., 2000; Liu et al., 2013).

### Transfusion and vasoregulation

Mounting evidence indicates that RBC transfusion impairs HVD efficacy, although the mechanism is not fully elucidated (Bennett-Guerrero et al., 2007; Bonaventura, 2007; Reynolds et al., 2011). In addition to the direct effects of cell-free Hb upon NO bioavailability in plasma, transfusion-associated hemolysis may also impair endothelial NO production via release of arginase (which, via substrate depletion, constrains eNOS activity) (Donadee et al., 2011; Alexander et al., 2013). Moreover, processed/stored RBCs are 2- to 4-fold more avid NO scavengers than fresh RBCs (Stapley et al., 2012) and exhibit more pronounced inhibition of NO mediated vasodilation. Further, mounting evidence implicates stored RBC-derived free-Hb and Hb-rich RMPs in dampening normal NO bioactivity in the microcirculation, leading to physiologically significant HVD impairment (Chen et al., 2008; Donadee et al., 2011; Kim-Shapiro et al., 2011; Roback, 2011). The latter observation is further supported by *in vivo* data demonstrating RMP contribution to the initiation of vasooclusive crises in SCD (Camus et al., 2015).

### RMP impact upon vasoregulation

**Figure 7:**
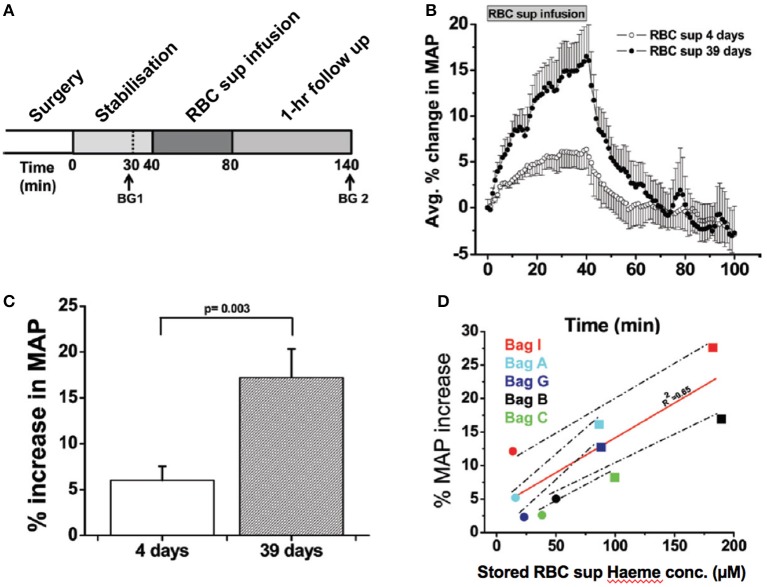
Vasoactivity of infused packed red cell supernatant/plasma. **(A)** Experimental time line for packed red cell supernatant infusions. Rats were stabilized for 30 min after surgery, and blood gasses were drawn as indicated (BG 1 and BG 2). Supernatant (1.6 mL) of packed red blood cells stored for either 4 or 39 days was infused for 40 min, after which the rats were followed for 1 h (*n* = 5). **(B)** Change in mean arterial pressure (MAP) over time after packed red blood cell (RBC) supernatant infusion and 60-min follow up. **(C)** Average percentage peak increase in MAP after infusion of packed RBC supernatants (RBC sup) (*p* = 0.003). **(D)** Correlation (solid line) between packed red blood cell supernatant haeme concentration and percentage increase in MAP after 40-min infusion of packed red blood cell supernatant stored for either 4 days (black solid circle) or 39 days (black solid square; R20.65). Each data point was obtained from a separate rat infusion experiment, in a different rat (2 groups of *n* = 5). All values are displayed as mean standard error of mean (SEM). Student *t*-test was used to compare the 2 groups of rats. Avg, average; conc, concentration. Adapted with permission from Donadee et al. (2011).

As discussed above, HVD is the principle physiologic reflex that maintains dynamic coupling between regional O_2_ delivery and tissue respiration, particularly during physiologic stress. RMPs appear to impair RBC-based HVD support of O_2_ delivery homeostasis, by: (1) preferential streaming in the cell free zone of the microcirculation and (2) acting as an NO sink. Specifically, animal studies demonstrate greater increase in mean arterial pressure (MAP) upon infusion of cell-free supernatants of longer stored RBC units (39 days) vs. fresher units (4 days) (Donadee et al., 2011; Figure 7); this increase correlates with the amount of extra-erythrocytic Hb (both free and in RMPs) in supernatants. Moreover, RMP half-life in this model was observed to be ~15–20 min, which is consistent with the time course for blood pressure changes that occur with infusion of stored RBC supernatants (Donadee et al., 2011). This is particularly important since (unlike cell-free Hb), RMPs are not bound by haptoglobin and may therefore contribute significantly to this phenomenon (Donadee et al., 2011).

## Summary

MPs are submicron particles that originate from varied cell populations, that form in response to a multitude of stimuli through cell membrane re-organization, blebbing and shedding. MPs have pleotropic effects upon vascular physiology and may serve as vehicles for inter-cellular communication. RMPs form spontaneously during the RBC lifespan, with content and cytoskeletal structure distinct from intact RBCs. RMP production accelerates during RBC storage due to associated biochemical changes: increased cytosolic calcium, ATP depletion, and increased potassium leakage. Moreover, RMP composition is affected by the trigger for their formation and by different storage conditions.

Proposed RMP biological effects include promotion of coagulation, immune modulation, and enhanced endothelial adhesion. Of particular importance, RMPs demonstrate significant NO trapping/consumption, disrupting regional matching between blood flow and tissue respiration that is essential to oxygen delivery homeostasis physiology. These effects have been demonstrated in animal models evaluating storage related RMPs, which appear to provoke an increase in systemic vascular tone and blood pressure following infusion of cell-free RBC unit supernatants. This effect is progressive with storage duration.

## Future directions

Our goals are to further elucidate the impact of RMPs on vasoregulation in critically ill subjects. We are currently examining the *in vivo* effects of RBC transfusions on hemodynamics, systemic vascular resistance and cardiac output, and tissue re-oxygenation during dynamic near infrared spectroscopy (NIRS), a novel non-invasive means to monitor hypoxic vasodilation (Creteur et al., 2009; Lipcsey et al., 2012). Concurrently, we are quantifying the peri-transfusion change in RMP burden and pharmacodynamics and the relationship to plasma vasoactivity which will be correlated with dynamic NIRS findings in humans.

## Author contributions

AS: performed experiments and analyzed data and drafted the manuscript. SR: performed experiments and analyzed data. AD: supervised experiments and edited the manuscript.

### Conflict of interest statement

The authors declare that the research was conducted in the absence of any commercial or financial relationships that could be construed as a potential conflict of interest.
